# Medical lectures upgraded: 11 hacks from comedy

**DOI:** 10.3205/zma001231

**Published:** 2019-05-16

**Authors:** Fabian Unteregger, Philipp Mayer

**Affiliations:** 1 Braintertainment GmbH, Herisau, Switzerland; 2 science-textflow, Winterthur, Switzerland

**Keywords:** presentation, humor, learning success, audience, techniques

## Abstract

We believe that medical lectures can be improved by considering techniques from comedy. Foremost, lecturers should educate their audiences. This works well, if lecturers have fun and entertain. In preparing the presentation, they should develop a storyline, try to surprise their audience, prepare to employ unexpected objects and carry out several test runs. During the presentation, lecturers should dare to use self-irony, appeal to students’ emotions, be factual and precise, serve the audience, keep it short and provide memorable opening and closing statements. Medical lectures should be both informative and entertaining.

## Zusammenfassung

Wir sind der Meinung, dass medizinische Vorlesungen durch Comedy-Techniken verbessert werden können. Grundlegendes Ziel ist es, Lerninhalte zu vermitteln. Das gelingt, wenn die Vortragenden Spaß haben und die Zuhörenden unterhalten. Beim Vorbereiten der Präsentation sollten die Vortragenden eine Storyline entwickeln, ihr Publikum (positiv) überraschen, unerwartete Objekte zur Illustration vorbereiten, und mehrere Probevorträge halten. Während der Präsentation sollten die Vortragenden Selbstironie wagen, Emotionen ansprechen, sachlich und präzise bleiben, ihrem Publikum dienen, sich kurz halten, und verblüffende Anfangs- und Schlussstatements abgeben. Medizinische Vorlesungen sollten beides sein: informativ und unterhaltsam.

## Introduction

Comedy is a form of entertainment intended to make people laugh [https://www.collinsdictionary.com/dictionary/english/comedy]. Comedians refine their material over dozens of shows so that only the best punchlines survive and are well embedded into a decent storyline. Some aspects that make good comedy can be directly transferred to medical lectures.

The aim of this article is to encourage and enable medical teachers to include appropriate humor in presentations [1], [2], [3], [4] and to use techniques from comedy. It is, however, not the idea to turn lecturers into comedians. Certain techniques, such as funny stories, funny comments, jokes and professional humor, work especially well in the teaching context [5]. Positive effects of humor in educational settings are widely accepted [5], [6], [7], because “humor appropriately used has the potential to humanize, illustrate, defuse, encourage, reduce anxiety, and keep people thinking” [5].

The eleven hacks from comedy described here sound simple, but in reality require practice and courage to apply all of them. The authors believe that if you try out one or more suggestions, more students will join and follow your presentations.

## Hacks

### The first hack is the foundation of every presentation

#### 1. Entertain

Lectures and presentations are unique opportunities to create entertaining moments for you and your audience [8]. Observe how much fun good speakers have while presenting. If we enjoy doing something and approach it with a positive attitude, we usually do it better [9], [10]. It is simply more pleasant to listen to somebody who enjoys presenting.

#### In preparing to present

##### 2. Use a storyline

Tell a story [11], [12], [13], [14], [15]. By far the most powerful technique is to share a personal story in the context of your lecture. In addition, the audience appreciates a roadmap to follow during a presentation [16]: it is true that “we are never tired, so long as we can see far enough” [https://www.brainyquote.com/quotes/ralph_waldo_emerson_397102]. In comedy, the context is provided first, to create a specific mindset. Only in such a predefined scenario can punchlines unleash their entire comic potential. In the academic context, you could share with the audience how you got into your field of research, or what lucky coincidence lead the way to a certain discovery or even what frustrated you.

##### 3. Surprise your audience

You will gain the audience’s attention by distinguishing yourself from your colleagues [17]. The essence of comedy is the punchline, which is nothing but a surprising twist in a storyline. In essence, every joke ends with a punchline. So why not approach your topic from a vastly different perspective? In cardiology, for example, anticoagulants play an important role in atrial fibrillation, which is seen in whales due to the size of their hearts. Hence, you could start the lecture by asking why, in spite of this fact, whale blood does not clot.

##### 4. Use surprising objects – but not too many of them

Even though a vast arsenal of presentation tools is available, building a presentation only around slides is a bad idea. Instead, include an unexpected object to illustrate your storyline. If, for example, you talk about the inhaling and exhaling mechanism of the lung, you might cut the bottom off a transparent plastic bottle, attach one balloon to the bottom and put another inside the bottle to illustrate the lungs and diaphragm. A neurology lecturer, having just returned from a conference in Asia, could bring some chop sticks along and tell the audience how much they struggled using them to show that the effects of neuroplasticity take some time. However, use objects briefly to visualize a mechanism.

##### 5. Field testing is key

It is helpful to present your material to one or more sets of audiences prior to the planned presentation [13], [14], [18], [19], [20]. Presenting to trusted colleagues can immediately confirm whether you are on the right track. In comedy and academia, appropriate timing of content delivery is acquired through field tests and experience. Before entering big stages, comedians always hone their skills and material on small stages to eliminate material which does not elicit laughter. So why not give your presentation to some colleagues over lunchtime? Honest criticism from their part should be actively sought to sharpen, polish and improve the lecture.

#### During the presentation

##### 6. Dare to use self-irony

**Figure 1:**
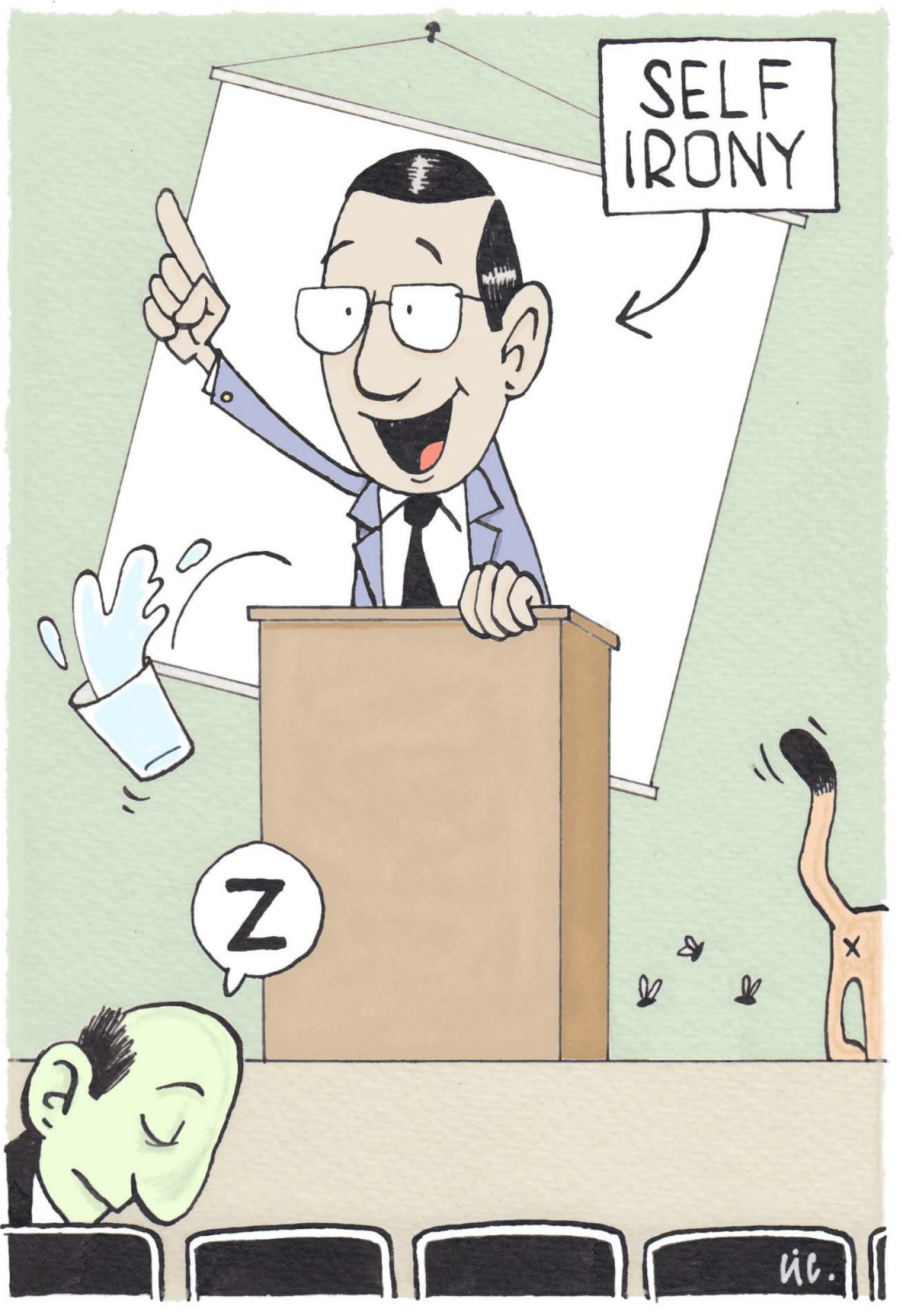
Dare to use self-irony and enjoy your show.

A personal account that is presented with humor and self-irony (see figure 1 (Fig. 1)) diminishes not only status differences between the lecturer and students, but also provides momentary relief from the formal atmosphere and the dry delivery of intellectually-demanding content [21]. Lots of comedians make fun of themselves which secures them the audience’s sympathy. In your lectures you could talk about exams you had to retake, experiments that failed and rejection letters you received. And the chop stick example mentioned in tip 4 comes in handy, too. 

##### 7. Dare to appeal to the students’ emotions

Students will instinctively appreciate a presentation filled with humorous, unembellished anecdotes and stories [15], [17]. It will require steady nerves to walk the fine line of appropriate informality, but it can also be said that no one will blame a speaker for attempting an entertaining presentation or lecture. Comedians always use material that speaks to the audience’s heart. They tell personal stories or speak of failure. This strategy can be directly applied to teaching. For instance, you could talk about how you thoroughly failed treating a patient (as long as this is not told as a joke as this could be seen as insensitive). Share this story and go through you own emotions you experienced back then.

##### 8. Be factual, specific, precise, and concise

Extract the essence and dare to discard the shell; less is more [13]. A clear and singular message appeals to the human mind. Furthermore, figurative language makes it more appealing. If content is too abstract, the students are lost - and their attention is difficult to regain. Yawning or fiddling with smartphones among the audience is evidence that this stage has been reached. In comedy, figurative language and an appropriate level of details are key. The same is true for medical lectures. If, for instance, you talk about a whale heart, you could inflate a balloon the size of a whale’s heart, to demonstrate that such big hearts have certain hemodynamic challenges.

##### 9. The students are the masters – you are their servant

Every audience is different. Even for a comedian experienced in performing live shows, the behavior of a new audience can be unpredictable. Still, every show provides valuable feedback. While lecturing, try to determine what draws the audience’s attention as early as possible. If you notice that a specific aspect is well received, dare to adapt and linger on the aspect, and then modify the remainder to stay within the time constraints [20]. You can even omit content since the students will be completely unaware. If you realize that they like a personal story you’ve shared when talking about a topic, do that more often!

##### 10. Keep it short

Brevity is refreshing. That’s why you loved reading this paragraph.

##### 11. Capture the students with mind-boggling opening and closing statements

The opening sentence creates the first impression and sets the tone [17], [20]. Once you have created a positive impression of your topic, the audience will be much more receptive to the rest of your content. The finale is equally important [17], [20]: express excitement and stimulate commitment. Referring to the cardiology example above, you may want to say: “But don’t forget: even if you are on an anti-blood clot regime, this does not mean that you can now dive like a whale!” Ultimately, this is what commits your message to memory. The final words may even generate laughter; this is where science and comedy meet.

## Author contributions

FU and PM both synthesized the literature and wrote the manuscript. 

Both authors contributed equally to this work.

## Competing interests

The authors declare that they have no competing interests.
